# Straining at a Gnat

**Published:** 1893-12

**Authors:** 


### STRAINING AT A GNAT.

There is a law which prohibits the sending of obscene literature or pictures through the United States mails, as is well known. It seems that the law designates as contraband pictures which display the sexual organs, or parts thereof, and certain other parts of the human body. The law was enacted, presumably, in the interest of morality; and doctors, accustomed all their lives to the anatomical and surgical illustrations of the text in medical works never dreamed of immorality in connection therewith, it is to be presumed, such idea as their being obscene or immoral never entered one's head; hence our readers will be surprised to learn, as we were, that one Anthony Comstock, connected with the United States mail service—a Puritan from Puritanville, no doubt,—sees great danger in the illustrations of medical works, and has "ruled" that they come within the purview of the act, and must be tabooed. The ubiquitous, smiling and pleasant little drummer for a surgical and gynecological operating table has been arrested, we understand, by a United States postoffice inspector, for exhibiting to doctors, in illustrating the uses of his table, certain cuts representing the manner of examining and operating in gynecological work. Under the head of "Purism," our St. Joseph contemporary works off a page or so of righteous indignation forninst the ruling, and against Comstock and the inspector aforesaid, individually and collectively.

This ruling has never been excelled for absurdity and pig-headedness in the annals of the postoffice department. It has never been equaled by any piece of absurdity we ever heard of,—unless it was that of the Boston lady who had pantalets put on her piano legs.

The decision of Mr. Comstock may well be compared to the

Scriptural figure of straining at a gnat; the corollary, the swallowing of the camel, is found in the fact that pictures really obscene, and accompanied by a text which, if not within itself immoral, is highly suggestive, are permitted to go through the mail, undisturbed, and at pound rates. The newspapers, some of them, are full of them. There is one advertisement in particular, which we think highly objectionable, and which we see not only in newspapers, but in certain high class magazines that go into the families of the best people. It is the picture of a man and a woman in nearly a nude state, holding above their heads a nude baby. In heavy capitals it is announced that "*the triumph of love is fruitful marriage*," and it tells of "a book for men only," which book will be sent through the mails, sealed, upon receipt of the price, and in it, it is promised that the book will tell all about love and the fruitful marriage, as well as give directions for developing "certain organs" which from abuse or what not, have not attained the full regulation size.

We took occasion to cut out this advertisement and submit it, along with an advertisement of "Tansy Wafers," to Bro. Wannamaker, when he was Postmaster General, and asked if such things were not contraband. The reply we received was in substance, and about to the effect, that while they were, in fact, obscene, and ought to be barred, yet technically they did not come within the scope of the prohibition, and nothing could be done to stop it. We were further consoled with the assurance that "Congress is now at work upon a bill which it is hoped will have the effect of excluding such publications," and so forth, and so forth.

If this is not straining at a gnat and bolting a robust camel, we do not know where an illustration of the simile could be found. Verily, we have a virtuous administration and a supersensitive postoffice department, whose modesty would be shocked at the illustrations in Gray's Anatomy! They should put petticoats on the "Greek Slave," and pinafores on the "Graces."

